# Differences in aqueous humor protein profiles in patients with proliferative diabetic retinopathy before and after aflibercept treatment

**DOI:** 10.1186/s12886-024-03292-1

**Published:** 2024-01-22

**Authors:** Tan Wang, Huan Chen, Xiaolan Du, M. M. Bintao Qiu, Ningning Li, Hanyi Min

**Affiliations:** 1 Department of Ophthalmology, Peking Union Medical College Hospital, Chinese Academy of Medical Sciences & Peking Union Medical College, No 1 Shuai Fu Yuan, Dongcheng District, Beijing, 100730 China; 2 Key Laboratory of Ocular Fundus Diseases, Chinese Academy of Medical Sciences & Peking Union Medical College, Beijing, 100730 China; 3 Medical Research Center, Peking Union Medical College Hospital, Chinese Academy of Medical Sciences, Beijing, 100730 China; 4 Operating Room, Peking Union Medical College Hospital, Chinese Academy of Medical Sciences & Peking Union Medical College, Beijing, 100730 China; 5 Department of Ophthalmology, Aier Eye Hospital, Tianjin University, Nankai District, Kanfu Road No. 102, Tianjin, China

**Keywords:** Proliferative diabetic retinopathy, Proteomics, Aflibercept, LC-MS/MS

## Abstract

**Purpose:**

To investigate the changes in aqueous humor (AH) protein profiles before and after intravitreal aflibercept (IVA) treatment in patients with proliferative diabetic retinopathy (PDR).

**Methods:**

5 PDR patients provided 10 samples of AH before and after IVA treatment (pre-group vs. post-group). Proteins were identified using liquid chromatography-tandem mass spectrometry. Then, bioinformatics was employed to investigate the functional significance of differentially expressed proteins (DEPs) and hub proteins.

**Results:**

A total of 16 DEPs were identified, consisting of 8 downregulated proteins and 8 upregulated proteins. Bioinformatics analysis indicated that the most significantly enriched biological process was “blood coagulation, intrinsic pathway.” The most significantly enriched signaling pathway was “complement and coagulation cascades.” HBB, HPX, VEGFA, and CA1 were identified as hub proteins for IVA treatment.

**Conclusions:**

Together with the downregulation of the intravitreal vascular endothelial growth factor level, IVA may also change the AH protein composition in PDR patients, with DEPs involved in the blood coagulation, intrinsic pathway, complement, and coagulation cascades. IVA treatment may protect against PDR by regulating HBB, HPX, VEGFA, and CA1 expression.

**Supplementary Information:**

The online version contains supplementary material available at 10.1186/s12886-024-03292-1.

## Introduction

Diabetic retinopathy (DR) is a complex and multifactorial disease characterized by pathophysiological mechanisms triggered by hyperglycemia. These mechanisms involve intricate interactions of genetic and epigenetic factors, heightened production of free radicals, formation of advanced glycation end products, advanced glycosylation end products, inflammatory factors and vascular endothelial growth factor (VEGF). Notably, angiogenesis plays a pivotal role in DR, particularly in proliferative diabetic retinopathy (PDR). As a consequence, the advent of intravitreal anti-VEGF therapy in the past decade has significantly transformed the therapeutic landscape for retinal vascular disorders. This approach facilitates targeted VEGF blockade by direct delivery of the drug into the eye, thereby minimizing systemic side effects and presenting a promising avenue for effective treatment of DR [1, 2]. Owing to its demonstrated high efficacy and favorable safety profile, anti-VEGF therapy has emerged as the recommended first-line treatment for PDR in clinical practice [3]. Aflibercept, a frequently prescribed anti-VEGF medication, inhibits placental growth factor signaling in addition to VEGF [4, 5]. The extracellular binding domains of VEGF receptors 1 and 2 are linked to an immunoglobulin Fc domain to form the compound aflibercept [6, 7]. Notably, aflibercept has a higher affinity for VEGF (140 times higher) than ranibizumab, and its intermediate size of 115 kDa (vs. 48 kDa for ranibizumab) results in greater intravitreal binding activity over the course of one month [8–10].

Notwithstanding the wide adoption of intravitreal aflibercept (IVA) therapy for the treatment of PDR, the precise underlying mechanism of its therapeutic effect remains to be elucidated. In recent years, proteomics has witnessed significant advancements, particularly in mass spectrometry (MS) techniques, which enable the rapid and specific quantification of proteins and metabolites in various body fluids. This approach yields a wealth of quantitative information, offering insights into disease mechanisms and the identification of novel biomarkers with potential clinical utility for diagnosis and prognostication [11]. MS technology has facilitated comparative proteomic analysis of various human specimens, including aqueous humor (AH). Through these investigations, numerous crucial molecules implicated in diverse diseases have been identified. These molecules hold the potential to serve as molecular targets for novel drug design or as disease-specific biomarkers, offering valuable insights for studying disease mechanisms, enabling early diagnosis, assessing therapeutic efficacy, and predicting prognosis. Such findings lay a solid foundation and rationale for advancing research in these areas.

Hitherto, no studies have compared the AH protein profile before and after IVA therapy in the same patient, which can contribute to exploring the mechanism underlying the effects of IVA treatment on PDR. To investigate the changes in AH protein profiles between PDR patients before and after IVA therapy, ten AH samples were obtained from five PDR patients before and after IVA treatment, and a proteomic method based on liquid chromatography-tandem mass spectrometry (LC-MS/MS) was utilized to analyze the AH samples. This study aims to contribute to a deeper understanding of the mechanism underlying the effects of IVA treatment on PDR.

## Methods

### Subjects

Five PDR patients provided 10 samples of AH before and after IVA treatment (pre-group vs. post-group) were enrolled in this study. All participants in the study provided their informed written consent. The study was conducted following the principles of the Declaration of Helsinki for biomedical research and was sanctioned by the Ethics Committee of Drug Clinical Trials, Beijing Union Medical College Hospital, Chinese Academy of Medical Sciences (FW-HXKT2018103102421S1). The following criteria were applied for enrollment: PDR was clinically diagnosed [12], and participants had no other ocular illnesses, pregnancy, or severe systemic problems (except for diabetes mellitus). Patients with prior ocular treatments such as photodynamic therapy, surgery, or intravitreal injections were excluded. Before the treatment, all patients underwent ophthalmic exams testing, including B-ultrasonography and biomicroscopy of the anterior and posterior segments, axial length measurement, intraocular pressure assessment, corneal endothelial cell counts, and best corrected visual acuity (BCVA) testing. This study is an own-pairing pre- and post-control study.

Due to the limited availability of studies directly comparing changes in AH proteomics before and after IVA treatment, the sample size for this study was determined by referencing a study that employed LC-MS/MS to investigate the proteomics of vitreous humor and AH in patients with PDR [13]. This previous study provided crucial insights into the proteomics of vitreous humor and AH in PDR patients. Although the purpose of that study differs, we considered it a relevant reference for determining the sample size due to the similarity in the proteomics method (LC-MS/MS) and the complexity of variables.

### Collection and preparation of samples

Patients received preventive topical levofloxacin application for three days after providing informed consent. Following the application of a topical anesthetic, the surgical site was sanitized, and patients received intravitreal injections of anti-VEGF medications (aflibercept 0.05 mg) at the superior temporal pars plana. After the injection, prophylactic application of the antimicrobial drop was continued for another three days. The collection of samples of each patient was conducted twice using a sterile 1-mL insulin injection syringe with a needle: first before anti-VEGF treatment and seven days later. Each sample was centrifuged at 13,000 g for 10 min at 4 °C, transferred to a 1.5-mL microcentrifuge tube, and stored at -80 °C until subsequent analyses.

A high-intensity ultrasonic processor (Scientz, Ningbo, China) was used to sonicate the AH samples three times on ice in 7 M Urea [Amresco 0568-1Kg, USA] + lysis buffer (2 M Thiourea [Sigma-Aldrich, USA] + protease inhibitors + 0.1% 3-[(3-Cholamidopropyl) dimethylammonio]-1-propanesulfonate [CHAPS]).

Centrifugation was used to remove the residual particles for 10 min at 12,000 g and 4 °C. Additionally, 10 µL of supernatant and the Bradford Protein Assay Kit (Thermo 23,236, USA) was utilized to quantify proteome. Then, using the modified filter-aided sample preparation (FASP) method, proteins were trypsinized [14, 15].

Briefly, 30 min of 60 °C incubation in 25mM dithiothreitol (Bio-Rad, USA) was followed by 10 min of 50mM iodoacetamide alkylation in the dark to reduce the lysate sample. The samples were loaded onto a 10 kDa cutoff ultrafiltration membrane (Sartorius, Germany) and then incubated with trypsin at a 1:50 enzyme-to-protein ratio overnight at 37 °C.

Three rinses in 50 mM triethylammonium bicarbonate buffer (TEAB; 300 mL; Sigma T7408, USA) were followed by 10 min of 12,000 g spinning on the samples. The manufacturer’s instructions for Ziptip C18 pipette tips were followed for peptide desalting.

After the C18 solid phase extraction column was activated and balanced with acetonitrile (CAN; Thermo A955-4, USA) and 2% ACN 0.1% formic acid (FA; Thermo A117-50, USA), the sample was loaded ten times by pipetting, followed by 2% ACN 0.1% FA desalination and elution in 50% ACN 0.1% FA. Subsequently, the acquired eluent was put in a rotating vacuum dryer and refrigerated at -80 °C.

In 0.1% FA, dried peptides were reconstituted, collected, and separated into samples with the same lysate quantities to create a data-independent acquisition (DIA) specialized library. The remaining samples were used with the Biognosys iRT kit, which also involved making a 10 iRT buffer and adding it to each sample at a 9:1 ratio.

### High-pH reversed-phase fractionation

Additional high-pH reversed-phase chromatographic separation of digest samples was performed. The separating of peptide mixtures in a 30 µg digest sample was accomplished using a reverse chromatography column (RIGOL, Beijing, China). Peptides were dissolved in mobile phase A (100 µL; 2% (v/v) ACN, 98% (v/v) ddH_2_O, pH 10), after which the mixture was spun down at 14,000 g for 20 min.

Then, to achieve stepwise elution in the column, the mobile phase B (98% (v/v) acetonitrile, 2% (v/v) ddH_2_O, pH 10) was introduced into the supernatants at a rate of 1 mL/min. Individual 15-minute eluant fractions were acquired using mobile phase B steep gradients.

### MS acquisition

We analyzed each sample with a volume of 1 µg using an instrument of Orbitrap Fusion™ Tribrid™ MS (Thermo Scientific) and a 150 μm×150 mm×1.9 μm internally produced analytical column. The following linear gradient settings were applied in a system of binary solvents with 0.1% FA in H_2_O (A) and 0.1% FA in ACN (B) as the solvents: 3–8% B/4 min, 8–22% B/65 min, 22–35% B/12 min, 35–90% B/4 min, 90% B/5 min.

Then, the eluents were introduced directly into the MS instrument. The capillary temperature and spray voltage were set to 320 °C and 2.3 kV, respectively. The whole MS scanning ranged from 300 to 1400 m/z. The MS operated with a resolution of 60,000 in top speed mode for 15,000 resolution MS/MS scans in under 3 s. For HCD, the normalized collision energy was set to 32% and an isolation window was set to 1.6 m/z. MS1 scans (automatic gain control (AGC) target 4e5 or 50 ms injection time) were carried out between 300 and 1300 m/z for DIA analysis, with a DIA segmentation resolution of 30,000 (AGC target 5e5; for injection time).

### Identification and quantification of proteins

Biognosys’ Spectronaut pulsar program was adopted to analyze DIA data [16]. Targeted data analyses utilized the default program settings, and dynamic iRT was used for types of retention time prediction. Local mass calibration and infinite scrambled decoy production were also used. Additionally, we applied an MS2-level interference connection to eliminate fragments while keeping ≥ 3 for measurement, using interference signals as a basis. The standard false discovery rate was set at 1%.

The software package’s ID picker algorithm, based on the parsimony principle, was utilized for proteomic inference. RAW photos were converted to the Spectronaut file format and calibrated using the worldwide spectral library’s retention time dimension for conducting spectral library-based studies. Next, the data were utilized for spectrum analysis without any additional recalibration depending on retention time. Evaluation of data-dependent acquisition (DDA) data was conducted using Proteome Discoverer 2.3 (Trypsin/P (Promega, V5111, USA), with two missed cleavages). Variable modifications and the fixed modifications were performed using methionine and acetyl (protein N terminal) oxidation, and Cysteine carbamidomethylation, respectively, in the search criteria. Initial mass tolerances were set at 10 ppm and 0.02 Da for precursor and fragment ions, respectively [17]. Biognosys iRT peptides fasta (uploaded to the public repository) and UniProt human (UniProt human 73,940 20,190,731 iRT.fasta) served as references for DDA data searches.

### Identification of differentially expressed proteins (DEPs)

Using a matched-samples t-test, protein expression differences were assessed following median normalization to reduce experimental bias. *P* < 0.05 and|Log_2_fold-change| > 0.58 were standard of statistically significant DEPs. Data normalization and identification of DEPs were carried out using the “Wu Kong” platform (URL: https://www.omicsolution.com/wkomics/main/) [18].

### Pathway and process enrichment analysis of DEPs

Gene Ontology (GO) analyses, including the biological process (BP), cell components (CC), and molecular function (MF), were conducted to unveil the characteristic biological attributes of DEPs [19]. The biological signaling pathway information among the DEPs was determined through Kyoto Encyclopedia of Genes and Genomes (KEGG) analysis [20].

The program packages employed for analyzing GO terms and KEGG pathways included the ClusterProfiler V3.14.0 [21], Pathview V1.36.0 [22], and the Goplot V1.0.2 package [23] within the R software statistical analysis platform. Significance was determined as *P* < 0.05 and a q-value < 0.05.

### Protein-protein interaction networks development and identification of hub proteins

The protein-protein interaction networks (PPI) network of the differentially coexpressed proteins was constructed using the Search Tool for the Retrieval of Interacting Genes (STRING) [24]. Cytoscape was employed to visualize the network of DEPs (with combined score > 0.15) [25]. The degree, maximal clique centrality algorithm (MCC), edge percolated component (EPC), and maximum neighborhood component (MNC) algorithms were calculated to identify hub proteins from the PPI networks [26]. We intersected the results of the four algorithms and selected the top five nodes with the highest degree, EPC, MCC, and MNC scores. It was hypothesized that the proteins overlapping across these algorithms represented hub proteins associated with aflibercept.

## Results

### Demographic characteristics and identification of DEPs

**Fig. 1:**
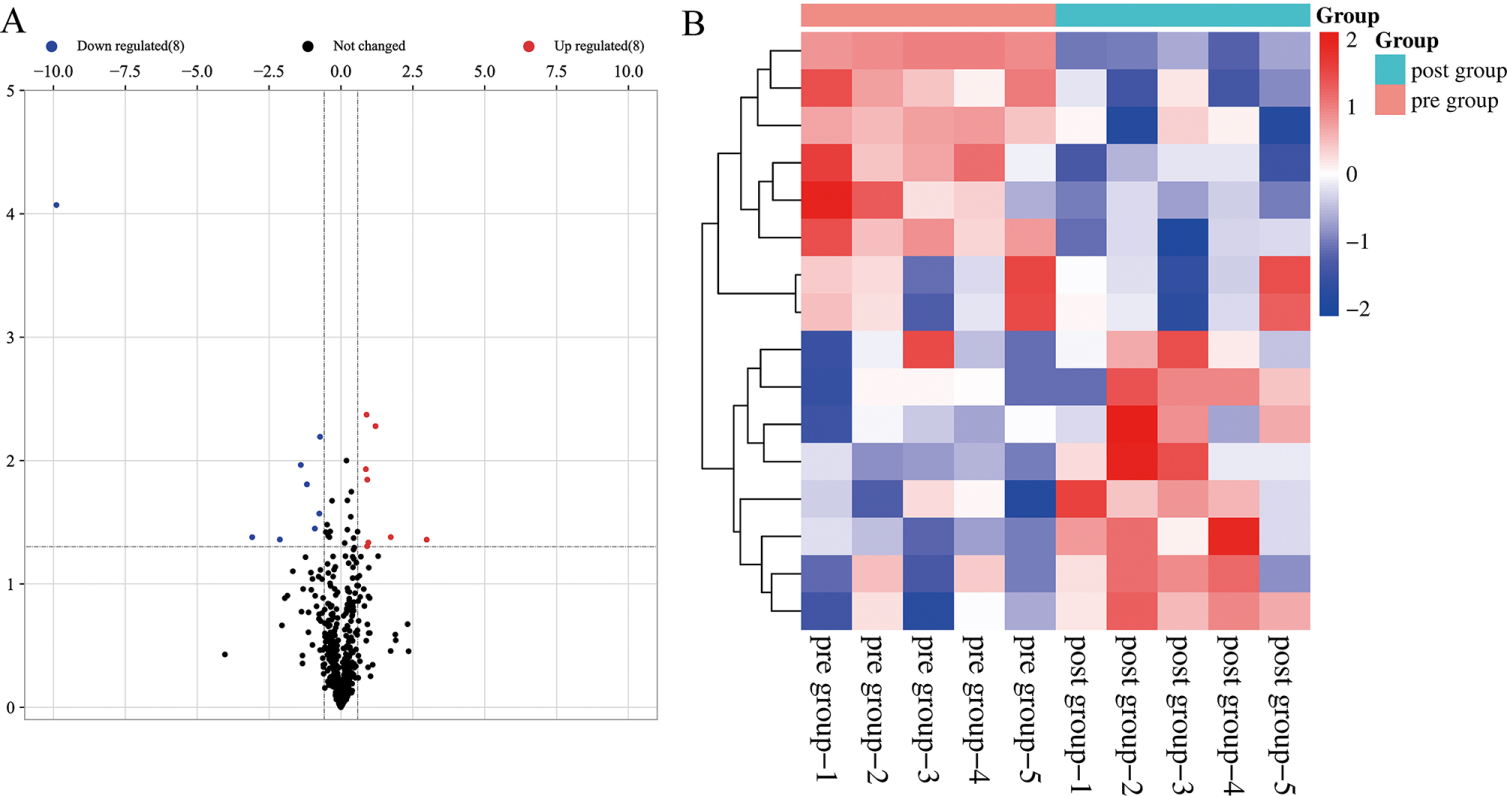
Differentially expressed proteins analysis. (**A**) Valcano plot. The x-axis represents the Log_2_fold-change, which indicates the magnitude of change in expression levels between two groups (before and after treatment), and the y-axis represents the statistical significance (the negative log_10_ of the p-value). Symbols in red represent upregulated proteins, and blue represents downregulated proteins. (**B**) Heatmap. Each row represents a protein, and each column represents a sample. The color of each cell in the heatmap indicates the relative expression level of a protein in a specific sample. The color scale represents the magnitude of expression, with higher expression levels represented by red color and lower expression levels represented by blue color

Clinical characteristics of patients, including preoperative eye examination results, are shown in Table 1. The study included 5 PDR patients with a mean age of 53.0 ± 8.7 years. A large-scale LC-MS/MS analysis was conducted, identifying 874 unique proteins (Table S1). Following filtering based on a 0.5 missing ratios in each group and subsequent imputation using the k-Nearest Neighbor method (k = 5), a total of 16 statistically significant DEPs were identified from a pool of 563 common proteins. These included 8 upregulated and 8 downregulated DEPs (Table S2, volcano plot in Fig. 1A, and heatmap in Fig. 1B).

**Table 1:** Baseline characteristics of subjects included in the analysis^*^

Variables		PDR patients (*n* = 5)
Age, y		53.0 ± 8.7
Male gender (%)		3 (60.0)
Right Eye (number (%))		5 (100.0)
DM history, y		13.0 ± 8.0
BCVA (LogMAR)		1.5 ± 1.0
Axial length (mm)		22.7 ± 1.0
IOP (mmHg)		16.5 ± 4.0
Staging of PDR	IV	1 (20.0)
	V	2 (40.0)
	VI	2 (40.0)

^*^Quantitative data and qualitative data are expressed as the mean ± SD and number of people (%), respectively. PDR, proliferative diabetic retinopathy; DM, diabetes mellitus; BCVA, best corrected visual acuity; IOP, intraocular pressure

### GO enrichment analysis

799 GO words related to all 16 DEPs were discovered (Table S3). In addition, the number of DEPs based on GO secondary function annotation was calculated. In the BP group, DEPs were dominantly enriched in blood coagulation, intrinsic pathway (3 proteins), protein activation cascade (3 proteins), blood coagulation, fibrin clot formation (3 proteins), angiogenesis involved in wound healing (3 proteins), regulation of humoral immune response (4 proteins), receptor-mediated endocytosis (5 proteins), humoral immune response mediated by circulating immunoglobulin (4 proteins), VEGF signaling pathway (3 proteins), humoral immune response (5 proteins), and complement activation (4 proteins).

DEPs in the CC group were primarily enriched in blood microparticle (5 proteins), immunoglobulin complex, circulating (3 proteins), endocytic vesicle lumen (2 proteins), immunoglobulin complex (3 proteins), collagen-containing extracellular matrix (4 proteins), external side of plasma membrane (3 proteins), primary lysosome (2 proteins), azurophil granule (2 proteins), tertiary granule (2 proteins), and haptoglobin-hemoglobin complex (1 protein).

The MF group DEPs were serine-type exopeptidase activity (2 proteins), immunoglobulin receptor binding (3 proteins), antigen binding (3 proteins), serine-type peptidase activity (3 proteins), serine hydrolase activity (3 proteins), aminopeptidase activity (2 proteins), transmembrane receptor protein tyrosine kinase activity (2 proteins), transmembrane receptor protein kinase activity (2 proteins), exopeptidase activity (2 proteins), and protein tyrosine kinase activity (2 proteins).

**Fig. 2:**
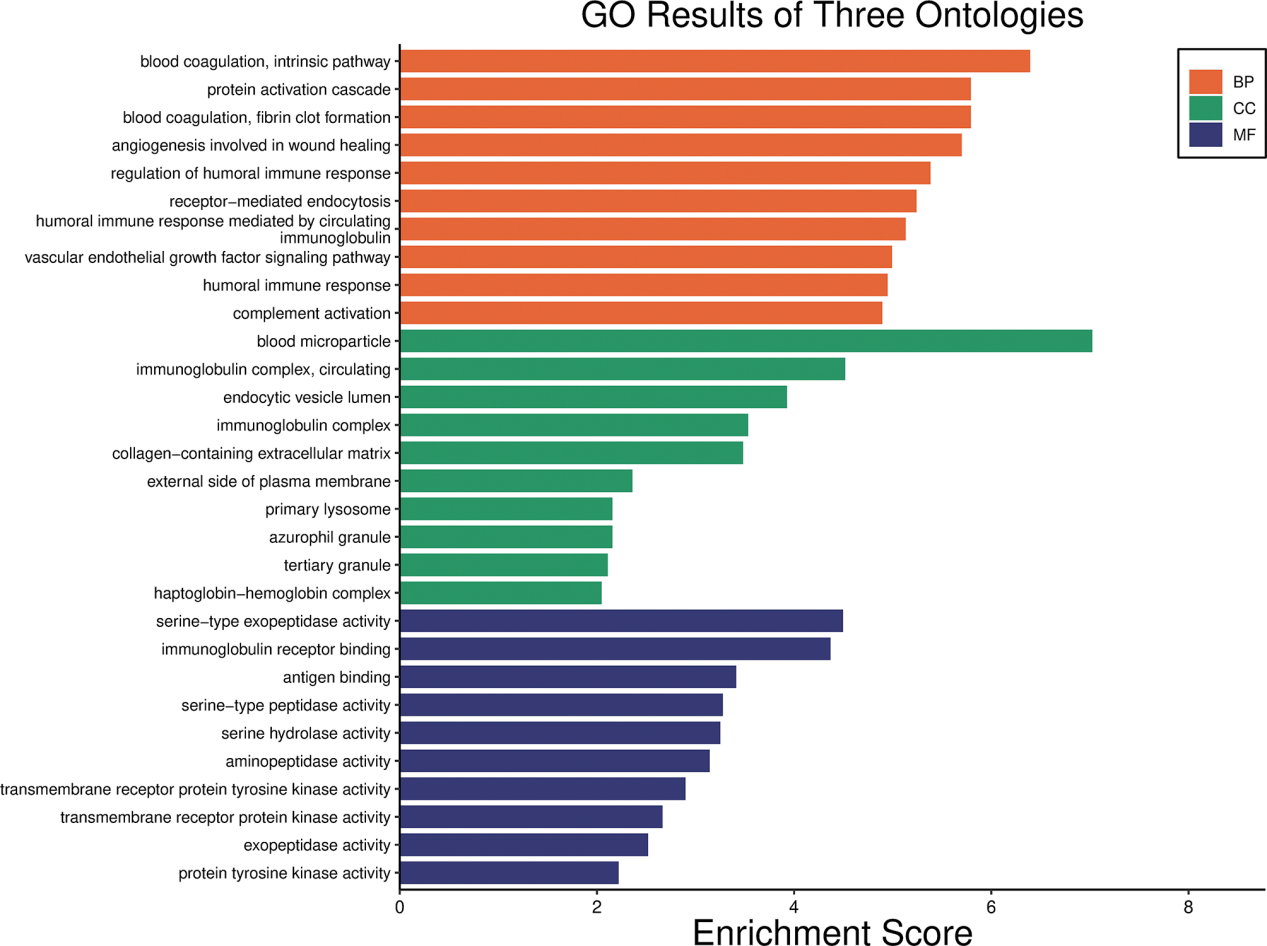
Gene Ontology (GO) enrichment analysis of differentially expressed proteins. The x-axis represents the enrichment score, and the y-axis shows the top 10 GO terms of molecular function, biological function, and cell composition. MF, molecular function; BP, biological processes; CC, cell composition

Enrichment analysis of DEPs was performed using the Fisher’s exact test (*P* adjust < 0.05) to determine the overall functional enrichment characteristics of all DEPs and to find the most significant enriched GO terms. The most significant enrichment of the BP term, MF term, and CC term was “blood coagulation, intrinsic pathway”, “blood microparticle” and “serine-type exopeptidase activity”, respectively (Fig. 2).

### KEGG pathway enrichment analysis

**Fig. 3:**
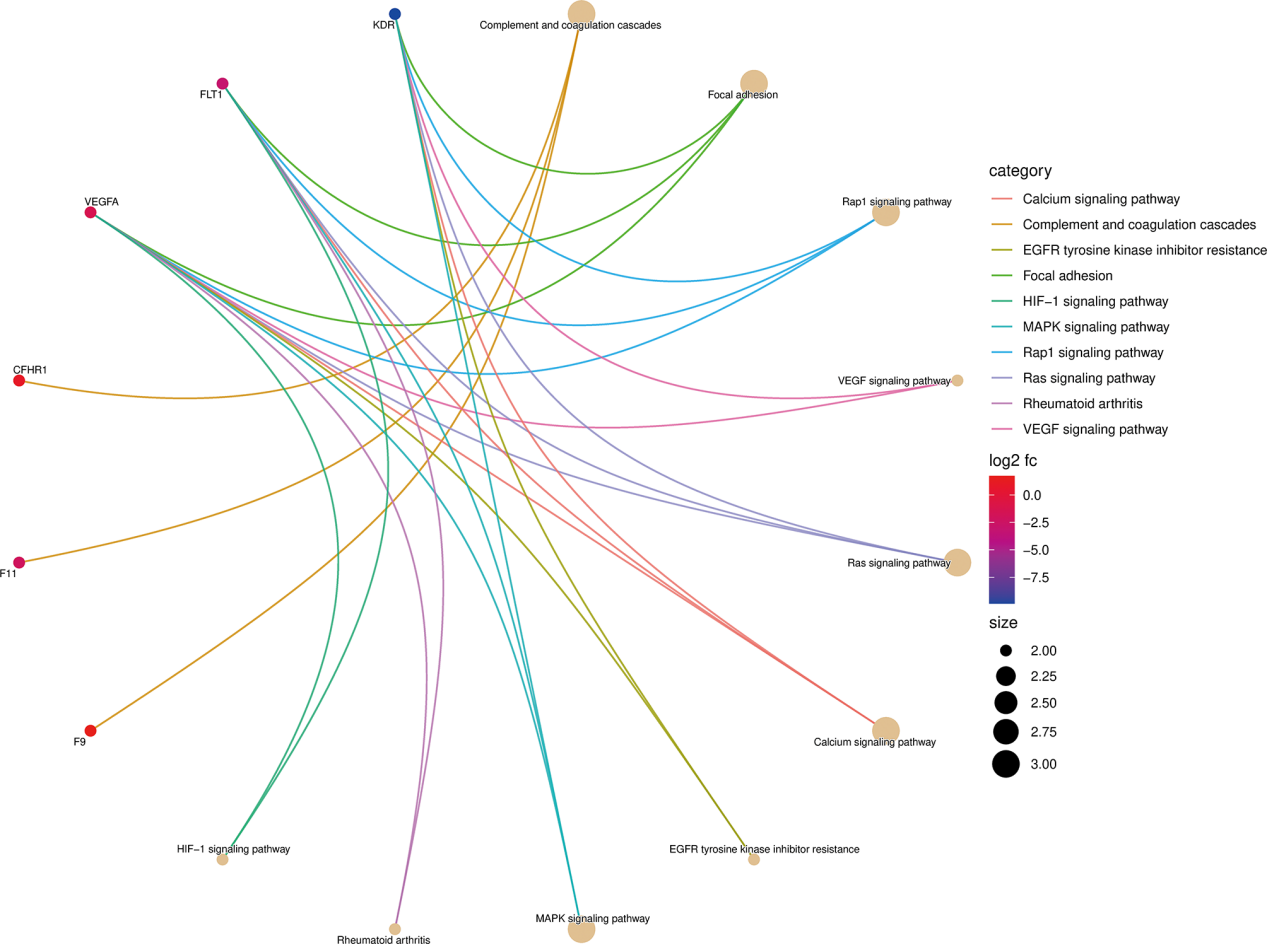
Kyoto Encyclopedia of Genes and Genomes (KEGG) pathway analysis of differentially expressed proteins. Symbols of differentially expressed proteins are presented on the left side of the graph. Symbols in red represent upregulated proteins, and blue represents downregulated proteins. Proteins involved in the KEGG pathways are indicated by colored connecting lines. The circle size represents the protein count enriched in the pathway

Figure 3 displays the outcomes of KEGG enrichment analysis on DEPs, indicating their primary enrichment in specific pathways: Complement and coagulation cascades (3 proteins), Focal adhesion (3 proteins), Rap1 signaling pathway (3 proteins), VEGF signaling pathway (2 proteins), Ras signaling pathway (3 proteins), Calcium signaling pathway (3 proteins), EGFR tyrosine kinase inhibitor resistance (2 proteins), MAPK signaling pathway (3 proteins), and Rheumatoid arthritis (2 proteins).

Furthermore, the Fisher’s exact test (*P* adjust < 0.05) revealed that the most significant enrichment was “complement and coagulation cascades”, followed by “Focal adhesion”, “Rap1 signaling pathway” and “VEGF signaling pathway” (Figs. 3, S1).

### Protein networks

**Fig. 4:**
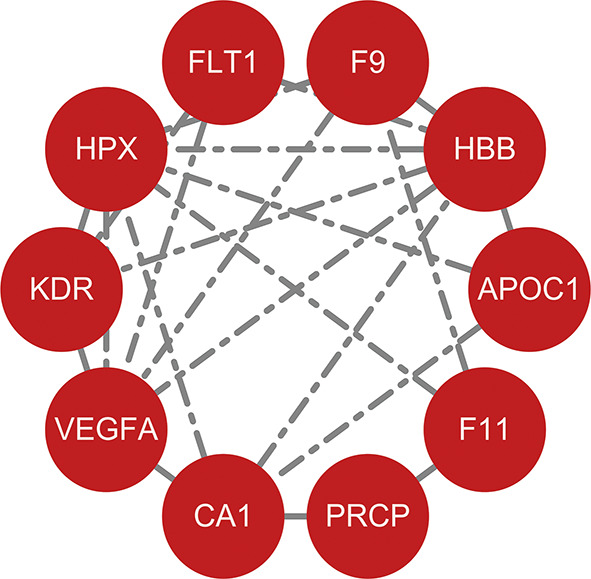
Protein-protein interaction network of differentially expressed proteins

To better understand the relationship between DEPs, we utilized the STRING database for PPI analysis. The PPI can be classified as known interaction (curated databases and experimental determination from literatures), predicted interaction (gene-neighborhood, gene fusion, and gene co-occurrence), or others (text mining, co-expression, and protein homology). Among the 16 DEPs, 10 (62.5%) proteins were found to interact with other proteins (Fig. 4).

### Hub proteins analysis

The degree, EPC, MCC, and MNC scores of DEPs were calculated using the CytoHubba plugin. We then selected the ten proteins with the highest scores in each algorithm and took the intersection of the four groups to improve the reliability of hub proteins. Finally, Hemoglobin subunit beta (HBB), hemopexin (HPX), vascular endothelial growth factor A (VEGFA), and carbonic anhydrase 1 (CA1) were considered to be hub proteins (Table 2).

**Table 2:** The degree, EPC, MCC and MNC scores of hub proteins

Proteins	MCC	MNC	Degree	EPC
HBB	30	7	7	6.45
HPX	26	7	7	6.388
VEGFA	24	6	6	6.194
CA1	13	4	5	5.868

MCC, maximal clique centrality algorithm; MNC, maximum neighborhood component; EPC, edge percolated component

## Discussion

In the present study, a total of 16 proteins were significantly differentially expressed in the AH of patients with PDR before and after IVA treatment. Our study employs a paired sample strategy to investigate. The most significantly enriched BP, according to bioinformatics analysis, was " blood coagulation, intrinsic pathway.” The most significantly enriched signaling pathway was “complement and coagulation cascades.” Thus, the therapeutic effects of IVA in PDR involve both VEGF decrease and modulation of the signaling pathway.

PDR is a severe complication of diabetes that can lead to vision loss if not managed effectively. Aflibercept is an anti-VEGF medication commonly used to treat PDR by reducing abnormal blood vessel growth in the retina. The study aims to explore how the protein composition in the AH, the fluid that nourishes the eye, changes in response to aflibercept treatment. By investigating these alterations, researchers can gain insights into the underlying mechanisms of aflibercept’s action and its impact on the disease progression.

The findings of this research have the potential to bring several benefits. Firstly, it may offer valuable information to clinicians, helping them understand the molecular changes that occur in the eye following aflibercept treatment. This knowledge can aid in refining treatment strategies and improving patient outcomes. Secondly, the identification of specific proteins that are affected by aflibercept treatment could serve as potential biomarkers for monitoring the therapeutic response and predicting treatment efficacy in PDR patients. These biomarkers may facilitate early detection of treatment non-responders or guide individualized treatment plans. Moreover, the research may uncover novel protein targets that play crucial roles in the pathogenesis of PDR. This could lead to the development of new therapeutic approaches and medications to complement or enhance the effects of aflibercept. Overall, this research has the potential to advance our understanding of the molecular changes associated with aflibercept treatment in PDR patients. By shedding light on the protein profiles in the AH, it may pave the way for more personalized and effective treatments, ultimately benefiting individuals affected by PDR.

The pathophysiology of DR is believed to involve the contact activation of the intrinsic coagulation system pathway, along with plasma kallikrein-induced kinin generation [27]. Proteomic studies of vitreous humor and AH have reported a link between the role of the complement and coagulation system and PDR [28, 29].

The five proteins with the highest scores in each algorithm for DEPs were selected, including the degree, EPC, MCC, and MNC algorithms. The intersection of the results of the four groups was analyzed to determine four proteins (HBB, HPX, VEGFA, and CA1) as hub proteins.

Hemopexin [30], which induces retinal pigment epithelium (RPE) cell death in vitro, is found to be overexpressed in the RPE of diabetic patients with DME. Hemopexin might function as a protective mechanism against the toxic effects of iron and mediated toxicity under normal physiological circumstances [31, 32].

Due to its ability to promote vascular endothelial cell migration and proliferation, Vascular Endothelial Growth Factor A (VEGFA) is critical for both healthy and pathological angiogenesis. It has been linked to the microvascular complication in type 1 diabetes and atherosclerosis. Numerous studies have shown that PDR is significantly exacerbated by VEGF [33, 34].

CA1 is implicated in modulating both vasoconstriction and vasodilation. The initial use of CA inhibitors to treat DR is based on an observational research that demonstrated the efficacy of systemically administered acetazolamide in treating macular edema [35]. Emerging research over the past few decades suggests that CA inhibitors in the treatment of DR [36].

The two different types of polypeptide chains that constitute adult hemoglobin are structured according to the HBA and HBB loci. The typical adult hemoglobin tetramer consists of two alpha chains and two beta chains. While no previous studies have reported an association with DR or aflibercept, the results of the PPI network of DEPs in this study indicate that HBB was linked to various DEPs, suggesting its potential contribution to the therapeutic efficacy of aflibercept for PDR.

In a previous study performed by Xinping She et al [37], the vitreous protein profiles in 6 PDR patients were compared before and after receiving a full anti-VEGF loading dosage of ranibizumab. They concluded that intravitreal ranibizumab treatment might protect against PDR by promoting SPP1 expression through the “GnRH secretion” and “Circadian rhythm” signaling pathways. However, to the best of our knowledge, there has been no similar previous study of aflibercept. Therefore, we used paired samples to analyze the short-term effects of aflibercept on the AH proteome in patients with PDR.

There were some limitations to this study. Firstly, the sample size was small, potencially limiting the generalizability of the findings. Secondly, to validate the results of our investigation, wet-lab techniques such as western blots, ELISAs, multiple-reaction monitoring, or parallel-reaction monitoring MS should have been conducted. Thirdly, conducting in vivo studies would be preferable to ascertain the potential mechanism of aflibercept and the therapeutic effects of the proteins mentioned in the study. In the future, we will collect more pre- and post-treatment AH specimens from patients treated with IVA to specifically test for hub protein expression. This will allow us to verify the difference in expression between the pre- and post-treatment groups. Additionally, we plan to use knockout mice for the corresponding hub proteins, subject them to IVA treatment, and compare them to wild-type mice to observe the difference in retinal protein expression levels. Finally, we aim to investigate the potential of other antibodies targeting these proteins for the treatment of DR in mice and assess the effectiveness of such treatment.

In conclusion, this study identified 16 DEPs in the AH of paired pre- and post-treatment patients with PDR using proteomics methods. Preoperative IVA treatment appears to regulate not only the levels of VEGF but also the levels of proteins involved in the blood coagulation, intrinsic pathway and complement, coagulation cascades, and other process in the AH of PDR patients. Importantly, the HBB, HPX, VEGFA, and CA1 levels were altered and these proteins may be involved in the pathogenesis of PDR and the mechanism of IVA treatment. These findings highlight novel targets and pathways for the treatment of PDR and provide a deeper understanding of the mechanism underlying the effects of IVA treatment in PDR.

### Electronic supplementary material

Below is the link to the electronic supplementary material.

**Supplementary Material 1**: **Fig. S1**. Complement and coagulation cascades pathway

**Supplementary Material 2**: **Table S1**. The proteins quantified in aqueous humor samples of enrolled patients. **Table S2**. Differentially expressed proteins (DEPs) between two groups (|log2FC| > 0.58, unpaired two-sided t-test, p value < 0.05). **Table S3**. The enrichment analysis of GO biological processes for Differentially expressed proteins (DEPs)

## Data Availability

All data generated or analysed during this study are included in this published article and its supplementary information files, or via ProteomeXchange with identifier PXD039210.
