# Supplementary figures and images for: Differences in aqueous humor protein profiles in patients with proliferative diabetic retinopathy before and after aflibercept treatment
Source: BMC Ophthalmol. 2024 Jan 22;24:32. doi: 10.1186/s12886-024-03292-1 (PMC10801989; doi:10.1186/s12886-024-03292-1)

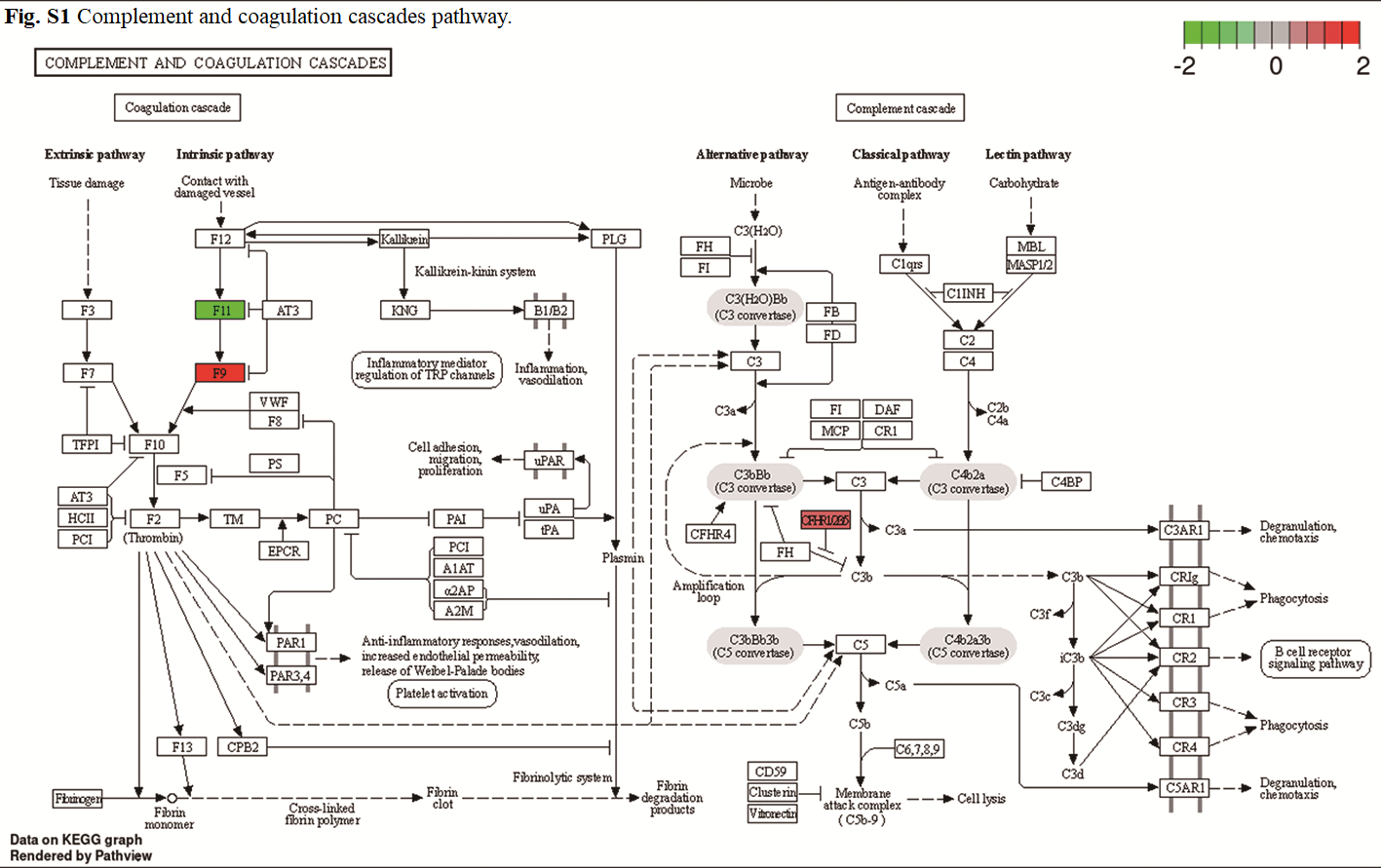

Supplement: Supplementary file 1 — Supplementary Material 1: Fig. S1. Complement and coagulation cascades pathway [file 12886_2024_3292_MOESM1_ESM.tif]
